# MR Neurography in Ulnar Neuropathy as Surrogate Parameter for the Presence of Disseminated Neuropathy

**DOI:** 10.1371/journal.pone.0049742

**Published:** 2012-11-14

**Authors:** Philipp Bäumer, Markus Weiler, Maurice Ruetters, Frank Staub, Thomas Dombert, Sabine Heiland, Martin Bendszus, Mirko Pham

**Affiliations:** 1 Department of Neuroradiology, Heidelberg University Hospital, Heidelberg, Germany; 2 Section of Experimental Radiology, Department of Neuroradiology, Heidelberg University Hospital, Heidelberg, Germany; 3 Department of Neurology, Heidelberg University Hospital, Heidelberg, Germany; 4 Center for Peripheral Nerve Surgery, Dossenheim-Heidelberg, Germany; Julius-Maximilians-Universität Würzburg, Germany

## Abstract

**Purpose:**

Patients with ulnar neuropathy of unclear etiology occasionally present with lesion extension from elbow to upper arm level on MRI. This study investigated whether MRI thereby distinguishes multifocal neuropathy from focal-compressive neuropathy at the elbow.

**Methods:**

This prospective study was approved by the institutional ethics committee and written informed consent was obtained from all participants. 122 patients with ulnar mononeuropathy of undetermined localization and etiology by clinical and electrophysiological examination were assessed by MRI at upper arm and elbow level using T2-weighted fat-saturated sequences at 3T. Twenty-one patients were identified with proximal ulnar nerve lesions and evaluated for findings suggestive of disseminated neuropathy (i) subclinical lesions in other nerves, (ii) unfavorable outcome after previous decompressive elbow surgery, and (iii) subsequent diagnosis of inflammatory or other disseminated neuropathy. Two groups served as controls for quantitative analysis of nerve-to-muscle signal intensity ratios: 20 subjects with typical focal ulnar neuropathy at the elbow and 20 healthy subjects.

**Results:**

In the group of 21 patients with proximal ulnar nerve lesion extension, T2-w ulnar nerve signal was significantly (p<0.001) higher at upper arm level than in both control groups. A cut-off value of 1.92 for maximum nerve-to-muscle signal intensity ratio was found to be sensitive (86%) and specific (100%) to discriminate this group. Ten patients (48%) exhibited additional T2-w lesions in the median and/or radial nerve. Another ten (48%) had previously undergone elbow surgery without satisfying outcome. Clinical follow-up was available in 15 (71%) and revealed definitive diagnoses of multifocal neuropathy of various etiologies in four patients. In another eight, diagnoses could not yet be considered definitive but were consistent with multifocal neuropathy.

**Conclusion:**

Proximal ulnar nerve T2 lesions at upper arm level are detected by MRI and indicate the presence of a non-focal disseminated neuropathy instead of a focal compressive neuropathy.

## Introduction

Diagnosis in peripheral neuropathies is often difficult given the vast number of potential compressive and non-compressive etiologies [1], so that a high percentage of cases remain classified as idiopathic [2], [3]. Ulnar neuropathy is one major diagnostic challenge due to the various potential lesion sites along the ulnar nerve. The most common form of ulnar neuropathy is due to a typical compression neuropathy at the elbow (UNE) of monofocal origin [4]. Localizing the lesion is of paramount diagnostic value but is further complicated by interindividual differences between the exit points of the various motor and sensory branches and by the possibility of selective fascicular lesions, which can mimic more distal lesions [5], [6], [7]. Therefore, diagnosis based on clinical examination and electrophysiological testing is frequently not conclusive regarding lesion localization and extension [8]. However, differentiating patients with a compression neuropathy from patients with more diffuse neuropathies of different etiologies is crucial for choosing the correct treatment.

MR Neurography (MRN) is an emerging method with high diagnostic accuracy in detecting focal neuropathies, such as UNE or Guyon’s canal syndrome (GCS) [9], [10]. Diagnostic criteria are T2 signal increase and caliber change. Other studies have confirmed the potential of MRN to detect the presence of neuropathy [11], [12], [13], [14], [15], [16], [17], but it has not been systematically examined to which extent the spatial lesion pattern information obtained by this technique is able to differentiate between pathologies of different etiologies. In true UNE the maximum of T2 lesion can exactly be localized to the cubital tunnel segment of the ulnar nerve at the elbow. Interestingly, we have occasionally observed that patients with clinical symptoms of ulnar neuropathy suspected to have UNE exhibit a more diffuse pattern of T2 lesions extending to the upper arm. It is hitherto unclear whether this atypical lesion extension is due to variability within the spectrum of entrapment neuropathies or whether it might instead suggest the presence of a more diffuse disease of different etiology. This would help to exclude a diagnosis of UNE, prevent unnecessary surgical decompression at the elbow, and initiate further diagnostic evaluation and treatment.

In this prospective investigation our goal was to determine the clinical significance of these disseminated ulnar nerve lesions. Specifically, we examined (i) whether the presence of atypical proximal ulnar nerve lesions correlated with other accompanying signs of diffuse neuropathy on MRI, namely the presence of lesions in other peripheral nerves, (ii) whether they correlated with unsatisfying outcome after decompressive surgery, and (iii) whether they would correctly predict the presence of a non-compressive neuropathy as assessed by clinical follow-up. We found that proximal ulnar nerve lesion extension is indeed indicative of an immune-mediated or hereditary instead of a focal compressive etiology.

## Patients and Methods

### Clinical and Demographic Patient Data

The study was approved by the institutional ethics board (University of Heidelberg ethics committee; S-057/2009) and written informed consent was obtained from all participants. Patients were examined at the Department of Neuroradiology of Heidelberg University Hospital, Germany between 01/2010 and 06/2012. The inclusion criterion was ulnar mononeuropathy of undetermined localization and etiology by clinical symptoms in the distribution of ulnar nerve motor and sensory function, as well as abnormal electrophysiological test results (parameters of ulnar nerve motor and sensory conduction). Exclusion criteria were any known inflammatory or other diffuse polyneuropathy such as distal symmetric polyneuropathy, or any evidence of C8 or Th1 nerve root compromise on cervical spine imaging.

A total of 122 patients underwent large coverage T2 sampling MR Neurography at the upper extremity because of clinically and electrophysiologically isolated ulnar mononeuropathy of unclear etiology. Coverage included the elbow region and upper arm, and in selected cases additionally the brachial plexus, forearm, and wrist depending on clinically suspected diagnosis. Of these 122 patients, 26 were identified with some degree of ulnar nerve lesion at the elbow but also an additional proximal lesion extension not normally seen in UNE. Three were excluded because retrospective re-evaluation of electrophysiological test results showed that slight alterations in median nerve conduction velocity were already apparent prior to MRN, and two were excluded because of altered MRN sequence parameters, leaving 21 patients in the study group.

Follow-up was conducted by reviewing medical records in the internal university archiving system and by standardized telephone interview with the referring physicians. Mean follow-up time was 9 months. Several of the patients had previously undergone elbow decompression for suspected UNE. In these, outcome after previous surgery for UNE was assessed by the physicians (F.S., T.D.) and defined as unsatisfying in case of missing improvement in strength of affected muscle groups by at least 1 grade on the MRC scale or persisting pain and paresthesia in the fourth and fifth finger.

Twenty patients (6 female, 14 male, age range 24–80 years, mean age 44) with unequivocal UNE by clinical and electrophysiological testing and MRN served as the first control group with typical monofocal neuropathy at the elbow (UNE). Twenty healthy subjects (9 female, 11 male, age range 25–70 years, mean age 42) served as the second, true negative control group. For healthy controls, upper extremity pain, hypesthesia or paresthesia or any history of polyneuropathy was excluded by interview (P.B., M.R.).

### MRN Imaging

Examinations were conducted using a 3 Tesla unit (Magnetom VERIO, Siemens AG, Erlangen, Germany). Subjects were examined in the prone position with the arm extended at the elbow placed in a knee 8-channel phased array coil. To avoid any significant artificial signal increase in a T2-w sequence related to the so-called Magic Angle effect, the longitudinal axis of the upper arm was aligned at an angle of ≤10° relative to the B_0_ field direction [18]. Two slice stacks were acquired at the upper arm and at the elbow with the following sequence parameters:

Transversal T2-w turbo spin echo TR/TE 6,980/52 ms, spectral fat saturation, slice thickness 3.0 mm, number of slices 45, interslice gap 0.3 mm, FoV 130×130 mm^2^, acquisition matrix 512×358, pixel spacing 0.254×0.254 mm^2^, number of excitations  = 3, acquisition time 7∶17 min.

### Image Analysis

Patients with proximal ulnar nerve lesions were identified by qualitative evaluation of images by consensus of two neuroradiologists (PB, MP) with more than four and seven years of training in MRN, respectively. Quantitative analysis was additionally performed on a SIEMENS Syngo Workstation (SyngoMMWP VE31A, syngVE32B) by two investigators (PB, MR) blinded to clinical and demographic patient data. Spatial registration was performed for each subject by referencing to the center of the osseous retroepicondylar groove (position = 0) as described previously [9]. Sixteen contiguous slices distally were incrementally labeled with positive numbers indicating their respective distance from the center (distance to center from 0 to +4.6 cm). Forty-four contiguous slices proximally were incrementally labeled with negative numbers indicating their respective distance from the center (distance to center from 0 to −14.2 cm). Slices at further proximal or distal positions were discarded and excluded from data analysis to ensure that positions subject to signal loss related to coil sensitivity profile in z-direction were not considered.

Intraneural T2-w contrast was evaluated on each image of both slabs at slice positions −14.2 through +4.6 cm by manually drawing the precise circumference of the ulnar nerve, and signal intensities within this intraneural region-of-interest (nROI) as well as the cross-sectional area were read out. Additional regions of interest were placed within non-denervated adjacent muscle (mROI), and in air to obtain the standard deviation in air (SD air) as a measure of image noise. Nerve contrast-to-noise ratios (CNR  =  (nROI – mROI)/SD air) and nerve-to-muscle signal intensity ratios, abbreviated SR, were calculated (SR  =  nROI/mROI).

### Statistical Analysis

Graphs mapping the proximal-to-distal course of nerve-to-muscle signal intensity ratio (SR), CNR, and area were plotted using Origin version 8.6G. Mean and maximum values in proximal (upper arm, corresponding to position −14.2 to −5 cm) and distal (elbow, corresponding to position −5 to +4.6 cm) imaging slabs were calculated for the three groups and were tested against each other for statistical significance using Student’s t-test, with a p value of <0.05 considered significant.

## Results

### MRN Findings

**Figure 1:**
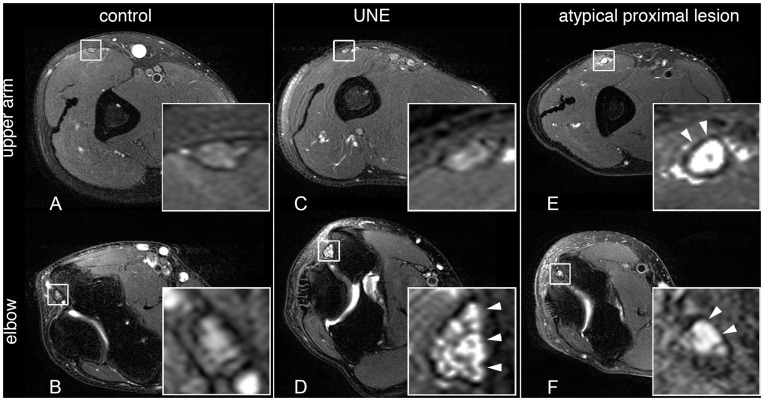
Typical imaging findings in healthy control, UNE and proximal ulnar nerve lesion. Upper row shows T2-w cross sections through upper arm, lower row through elbow. The first column [A and B] represents a healthy control with only slight ulnar nerve signal increase at the elbow. The second column is a typical UNE with inconspicuous ulnar nerve at upper arm level [C] and clear increase of signal and cross-sectional area at the elbow [D] (arrowheads). The third column shows a patient with atypical proximal lesion at upper arm level [E] as well as the elbow [F].

A total of 21 patients were included in the study with an unequivocal ulnar nerve lesion at the elbow but an additional proximal lesion extension not normally seen in UNE (figure 1).

These 21 patients with atypical lesion extension had varying clinical signs of ulnar neuropathy and initial suspect diagnoses (table 1).

**Table 1:** Patient demographics and clinical data.

No.	Age	Sex	Pain, paresthesia, hypesthesia	Subjective weakness	Atrophy	Previous decompressiveelbow surgery	Suspect diagnosis before MRI, reason for referral
**1**	57	M	Yes	Yes	Slight	Yes	persisting ulnar neuropathy after decompressive surgery
**2**	53	F	Yes	Yes	None	No	ulnar neuropathy lesion localization at arm
**3**	59	M	Yes	Yes	None	Yes	persisting ulnar neuropathy after decompressive surgery
**4**	64	M	Yes	Yes	None	No	ulnar neuropathy lesion localization, UNE vs. plexus
**5**	52	F	None	Yes	Moderate	No	unclear ulnar neuropathy with sonographical thickening of ulnar nerve proximal to ulnar sulcus DD perineurioma
**6**	56	M	Yes	None	None	No	suspected UNE
**7**	58	F	Yes	Yes	None	No	ulnar neuropathy lesion localization, UNE vs. GCS
**8**	62	M	Yes	Yes	Profound	Yes	persisting ulnar neuropathy after decompressive surgery
**9**	18	M	Yes	Yes	Moderate	No	ulnar neuropathy of unclear localization, plexus vs. elbow
**10**	46	M	Yes	Yes	Slight	Yes	persisting ulnar neuropathy after decompressive surgery
**11**	57	M	No	Yes	Slight	No	ulnar nerve lesion of unclear etiology, diabetic PNP
**12**	38	F	None	Yes	Moderate	No	UNE vs. motor neuron disease
**13**	47	M	None	Yes	Moderate	No	suspected UNE
**14**	48	M	Yes	Yes	Moderate	Yes	persisting ulnar neuropathy after decompressive surgery
**15**	47	F	Yes	Yes	Slight	Yes	persisting ulnar neuropathy after decompressive surgery
**16**	62	F	Yes	Yes	Moderate	Yes	ulnar neuropathy of unclear localization, elbow vs. plexus
**17**	39	M	Yes	Yes	Slight	No	suspected UNE
**18**	51	M	Yes	Yes	None	Yes	persisting ulnar neuropathy after decompressive surgery
**19**	33	F	Yes	None	None	Yes	recurrent ulnar neuropathy after decompressive surgery
**20**	58	M	Yes	None	None	No	ulnar nerve lesion of unclear etiology, lesion localization not possible by inching method
**21**	51	F	Yes	None	None	No	suspected UNE vs. C8 syndrome

**Figure 2:**
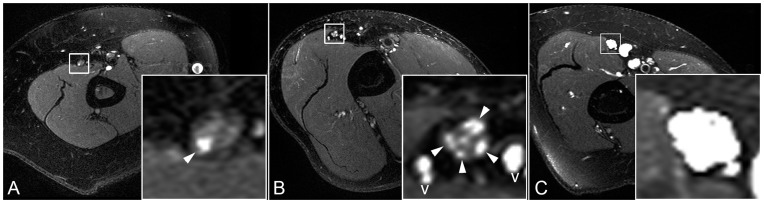
Spectrum of proximal ulnar nerve lesions. T2-w cross-sections through upper arm with ulnar nerve magnifications. [A] shows a restricted lesion involving only one individual fascicle, [B] shows multiple fascicular lesions (arrowheads) not to be confused with adjacent small vessels (v), [C] shows a whole-nerve lesion with severe caliber increase.

On MRN, the ulnar nerve showed varying degrees of neuropathy (figure 2). In several cases, not the entire cross-section of the nerve exhibited a signal increase. Instead, the pathologic T2 signal was confined to a single or only a few fascicles while the remaining fascicles appeared normal.

**Figure 3:**
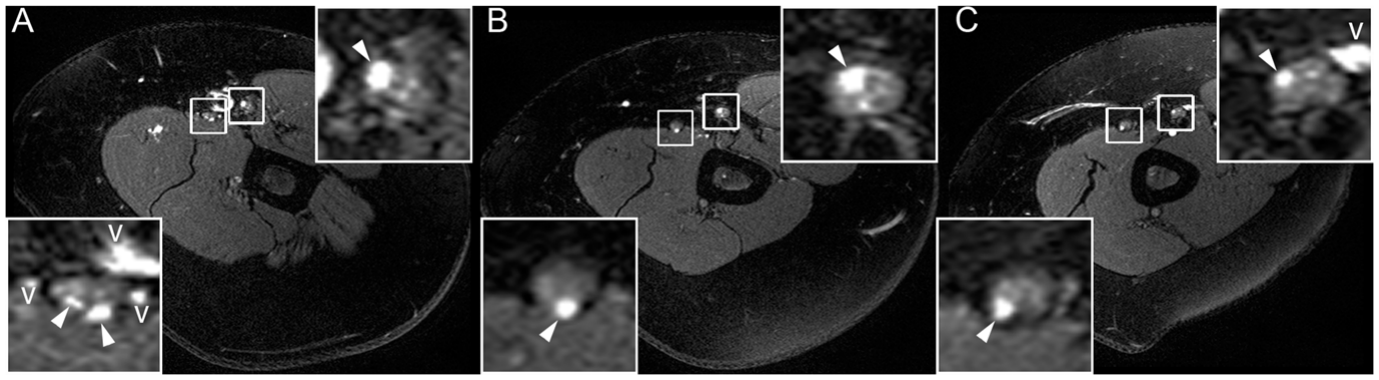
Continuous fascicular lesions. Lesions in one individual fascicle of both ulnar nerve and median nerve at upper arm level extending over a distance of at least 10 cm. [A] shows a T2-w proximal cross-section, [B] is at mid upper arm, [C] at a more distal position few cm above the elbow. Lower left magnifications show the ulnar nerve, upper right magnifications the median nerve. Fascicular nerve lesions (arrowheads) appear within the nerve, concomitant vessels (v) are adjacent to the nerve.

**Figure 4:**
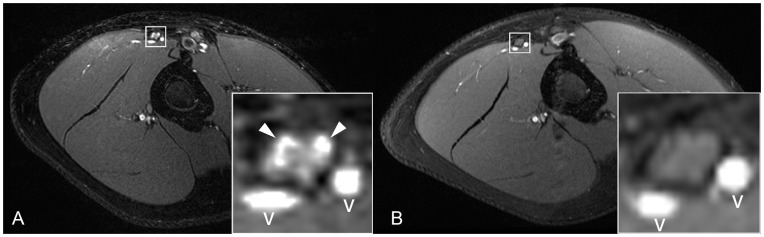
Differentiation of fascicular lesions versus small vessels. [A] T2-w image in combination with [B] contrast-enhanced T1-w image with fat-saturation at the same position allows discriminating fascicular lesions (arrowheads) from adjacent small vessels (v).

Occasionally, lesions were observed to continue along one individual fascicle for a larger distance while sparing all adjacent fascicles within the same nerve (figure 3). Small concomitant vessels could reliably be excluded by their tortuous course on axial slice-by-slice and eventual entry or exit from the epineurial vicinity. When in doubt, administration of contrast media excluded the presence of intraneural veins (figure 4).

Review of the clinical history of the 21 patients revealed that 10 (48%) had previously been operated for UNE with unsatisfying outcome. Three of these and another seven of 21 (48%) patients with atypical proximal lesion extension along the ulnar nerve additionally exhibited subclinical lesions in the median or radial nerve (one example shown in figure 3). When lesions in the median or radial nerve were detected, they were most prominently displayed at the upper arm (8 of 9), were generally less pronounced but still visible at the elbow (7 of 9), and were found below the medial epicondyle in only one case.

### Quantitative Evaluation

**Figure 5:**
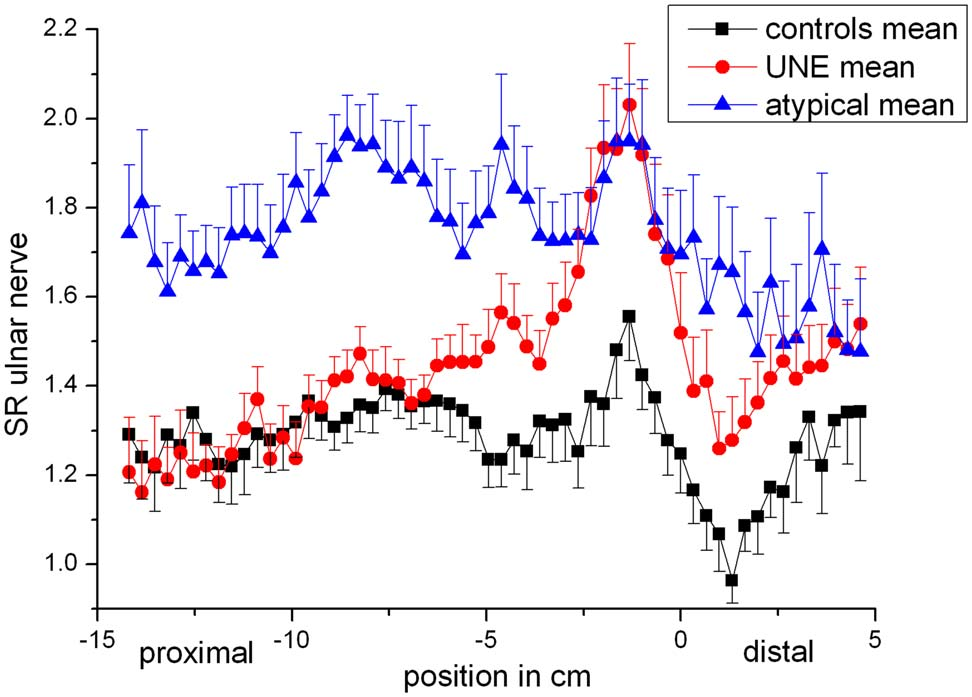
Ulnar nerve-to-muscle signal intensity ratios. Average values for the three groups (healthy controls, UNE, atypical proximal lesions) were calculated at each slice position and plotted. The patient group with atypical proximal ulnar nerve lesions had significantly elevated nerve-to-muscle signal intensity ratios at upper arm level, while UNE patients had a pathological signal increase at the elbow compared with only slight increase in healthy controls.

Our qualitative assessment of the ulnar nerve lesions was additionally tested by quantitative evaluation of nerve-to-muscle SI ratios. A cohort of 20 healthy subjects as well as 20 patients with typical UNE served as controls. The longitudinal proximal-to-distal mapping of the T2 signal intensity (SI) ratio between the ulnar nerve and adjacent healthy muscle tissue quantified and confirmed the qualitative reading: patients with atypical proximal lesion extension in the ulnar nerve showed an increased T2 nerve-to-muscle SI ratio ranging from the proximal upper arm to the elbow, as opposed to patients with typical UNE who had a peak at the elbow and only few cm proximal to it (figure 5). Healthy controls showed a spatially restricted increase at the retroepicondylar ulnar groove (ulnar sulcus) as described previously. CNR quantification yielded essentially the same results as the T2 nerve-to-muscle SI ratio (data not shown). The nerve cross-sectional area graph followed a similar course as the T2 nerve-to-muscle SI ratio (data not shown), but with less obvious relative differences since nerve diameter was not as frequently and as dramatically increased.

Mean values and maximum values of the ulnar nerve-to-muscle SI ratio at the upper arm and at the elbow were compared among the groups and tested for significance. The patient group with proximal lesions had significantly higher values than the UNE control group at the upper arm (mean 1.85±0.40 vs. 1.41±0.17, p<0.001; max. 2.38±0.48 vs. 1.67±0.19, p<0.001), but not at the elbow (mean 1.71±0.43 vs. 1.73±0.35, p = 0.28; max. 2.42±0.62 vs. 2.74±0.56, p = 0.67). Compared to healthy controls, the patient group with proximal lesions had significantly higher values both at the upper arm (mean 1.85±0.40 vs. 1.33±0.20, p<0.001; max. 2.38±0.48 vs. 1.61±0.21, p<0.001) and at the elbow (mean 1.71±0.43 vs. 1.32±0.19, p<0.001; max. 2.42±0.62 vs. 1.85±0.34, p<0.001). UNE patients had higher values than controls at the elbow (mean and maximum values p<0.001) but not at the upper arm (mean values p = 0.30, max. values p = 0.92).

**Figure 6:**
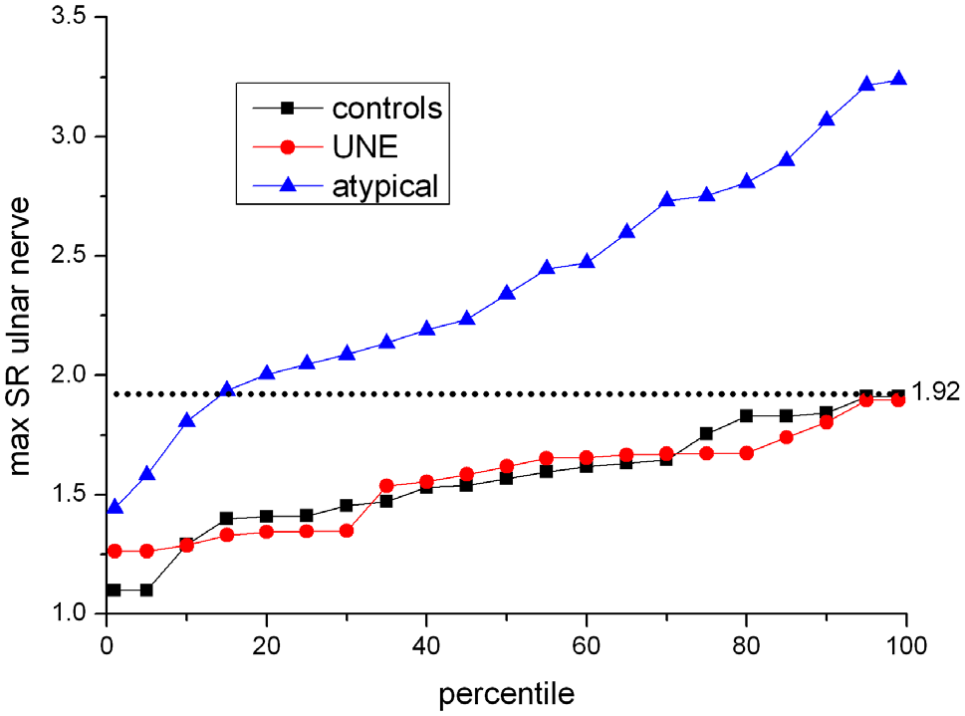
Percentile plot of maximum SR in proximal ulnar nerve. Percentiles for each group are plotted for maximum values of ulnar nerve-to-muscle signal intensity ratios at upper arm level. Dotted line indicates the most conservative cut-off value of 1.92 which completely excludes false-positives. The lowest value for the group with atypical proximal lesion corresponds to the case shown in figure 3.

Percentiles were calculated for maximum SR ulnar nerve values at the upper arm. Figure 6 shows the distribution of these maximum values. A conservative cut-off value to avoid false-positive results was found to be a SR of 1.92, with a sensitivity for the detection of proximal neuropathic lesions of 86% and a specificity of 100%.

### Follow-up

The atypical proximal lesion extension in the MRN examination prompted further diagnostic testing in several of these patients. Follow-up was available for 15 of 21 patients (table 2). Three patients had received a definitive diagnosis of inflammatory neuropathy. One patient was found to have a genetic neuropathy. In eight patients, diagnostic work-up had not yet established a definitive diagnosis but suspect diagnoses were consistent with a disease resulting in multifocal nerve lesions. In three patients, symptoms resolved without therapy.

**Table 2:** Results for patients regarding the three endpoints of (i) presence of other subclinical nerve lesions, (ii) previous elbow surgery, and (iii) results of clinical follow-up.

Patient	MRN lesions detected in other nerves	Previous surgery for presumed UNE	Follow-up
**1**	No	Yes	multifocal acquired demyelinating sensory and motor neuropathy (MADSAM)
**2**	Yes	No	Sensorimotor neuropathy of ulnar nerve at upper arm, suspected inflammatory origin without definitive confirmation yet, no improvement by steroid therapy
**5**	Yes	No	multifocal motor neuropathy (MMN)
**6**	Yes	No	complete recovery without surgery, no further diagnostics
**7**	No	No	idiopathic ulnar neuropathy with spontaneous partial recovery
**8**	No	Yes	mononeuritis multiplex with improvement after steroid therapy
**9**	No	No	complete recovery without surgery or definitive diagnosis
**10**	No	Yes	no improvement after probatory immunosuppressive therapy
**11**	Yes	No	multifocal motor neuropathy (MMN)
**12**	No	No	idiopathic plexusneuritis
**13**	Yes	No	hereditary motor and sensory neuropathy type II (HMSN II)
**14**	Yes	Yes	mononeuritis multiplex
**18**	Yes	Yes	cervical spondylarthropathy with myelopathy and subsequent decompressive spinal surgery
**19**	Yes	Yes	symptom progression to territories of median nerve and contralateral ulnar nerve, no definitive diagnosis yet
**21**	Yes	No	idiopathic plexusneuritis

## Discussion

This paper reports that the proximal-to-distal distribution of an ulnar nerve T2 lesion along the upper arm and elbow is a reliable diagnostic marker to differentiate focal and compressive ulnar neuropathy at the elbow from disseminated neuropathy. This diagnostic discrimination is clinically highly relevant because conventional clinical and electrophysiological tests can be insufficient in clearly identifying proximal lesions of the ulnar nerve, which often results in falsely localizing ulnar neuropathy to the elbow. False positive diagnosis of focal UNE carries the risk of unnecessary surgical decompression at the elbow and may withhold targeted therapy of disseminated proximal neuropathy. In this study, both MRI-based qualitative evaluation and quantitative comparison clearly differentiated patients with proximal lesions from healthy controls and from typical cases with UNE. This differential lesion pattern is of immediate diagnostic value by suggesting the presence of a disseminated instead of a non-compressive neuropathy for the following three lines of argument.

First, 48% of cases additionally displayed lesions in the median or the radial nerve or both. These were clinically silent at the time of examination and had not been detected electrophysiologically. Previous quantitative studies show that these signal abnormalities must be considered true neuropathic lesions [9], [18].

Second, another 48% of patients had already been operated at the elbow for UNE without satisfying improvement. Generally, only about 70% of patients after surgery for UNE report improvement or complete remission [19]. We are careful not to suggest that all of the remaining 30% of patients have a disseminated neuropathy of non-compressive etiology. Still, for some of them and in 10 of the patients we included in this study, the presence of a non-focal, disseminated neuropathy is a conceivable explanation for lack of improvement after surgery. Although surgery itself may rarely cause an inflammatory neuropathy [20], it is unlikely that all of these patients have persisting proximal nerve lesions as a consequence of local decompression at the cubital tunnel. Many other post-surgical patients do not exhibit this lesion pattern even when symptoms are persistent. In our group, three patients had both been operated and displayed neuropathic lesions in additional nerves at the upper arm. We consider it highly unlikely that, e.g., the deep proximal radial nerve within the spiral groove at the dorsal aspect of the humerus is affected many months after targeted compression of the ulnar nerve.

Third, clinical follow-up confirmed our suspicion of a disseminated neuropathy in several individuals. Diagnoses included inflammatory and genetic neuropathies, such as multifocal motor neuropathy and hereditary motor and sensory neuropathy type II. A larger number of patients in our study group were still diagnosed as mononeuropathy multiplex or other idiopathic ulnar neuropathy of undefined etiology at the time of follow-up. In the literature, around a quarter of patients with peripheral neuropathies examined at specialized centers remain without definitive diagnosis [2], [3]. Patients referred to an MRN exam are likely overrepresented in this subgroup since the method is normally a late link in the diagnostic chain.

Our results have potential implications for the management of ulnar neuropathy. An MRN exam covering the elbow and the upper arm and possibly including additional regions depending on the presentation of symptoms seems warranted if history and clinical/electrophysiological examinations are equivocal. Based on this analysis of the upper extremity, the most likely region to encounter neuropathic lesions in disseminated neuropathy by MRI seems to be the upper arm.

Further, in our study group, MRN seemed to be more sensitive in finding neuropathic lesions than any other employed methods. This was due to the study design which excluded any patients with pathologic clinical or electrophysiological findings in other nerves prior to MRI. It has not yet been systematically evaluated whether MRN is generally more sensitive than other methods in identifying neuropathy. Studies using ultrasonography have suggested that nerve enlargement already occurs in asymptomatic individuals and represents subclinical involvement [21]. Even more sensitive than nerve caliber is the presence of a T2 lesion on MRI [9], [10]. These arguments as well as our clinical experience suggest that MRI may be the most sensitive method for the detection of neuropathy.

One particular challenge in image evaluation is thresholding. For the MR sequence we employed, the calculated cut-off value of maximum SR at the upper arm for the differentiation between healthy and affected nerves was 1.92, with a sensitivity of 86% and specificity of 100%. This value can be considered to be very conservative since we used individual maximum and not average values to calculate it and chose a specificity of 100% so as not to include false-positive findings. Still, we would like to emphasize that in our experience lesions normally appear to the reader without the need to quantify signal intensity ratios in clinical routine.

As one additional interesting finding, we here report selective fascicular lesions for the first time. Partial traumatic lesions have been observed to cause a fascicular lesion pattern not affecting the entire nerve [22]. In individual cases, small intraneural nerve sheath tumors have been observed to mimic more distal lesions by selectively involving only one or a few fascicles [23], [24], [25]. Restricted affection of an individual fascicle by a more disseminated neuropathy has long been deduced by neurologists by clinical experience [7] but has to our knowledge previously not been confirmed by imaging.

There is no conclusive evidence in the literature that the parameters of nerve T2 signal intensity and caliber as employed in this study can reliably distinguish between the different etiologies of disseminated neuropathies. Functional techniques like DTI may in the future become helpful in the differential diagnosis by MRI, possibly by helping to distinguish axonal from demyelinating neuropathies.

This study comes with several limitations. First, the 21 cases represent a very heterogeneous group of patients with unclear ulnar neuropathy. Obviously, no diagnostic gold standard exists for this group. To counter this heterogeneity, we applied strict inclusion and exclusion criteria, and three patients were later excluded because retrospective reevaluation of electrophysiological findings already suggested median nerve affection. Second, a bias might have been introduced through the inclusion of previously operated patients. As discussed above, we cannot exclude that surgery itself may have been a factor for persistence of neuropathy in an individual case, but consider it highly unlikely that this should be the case in the majority of post-surgical patients. Third, follow-up could not be obtained for six of the patients. Among the 15 patients for whom clinical follow-up was available, reported diagnoses were of heterogeneous finality. While in some cases, diagnostic testing had come to a definitive conclusion, in other cases only descriptive diagnoses such as mononeuritis multiplex or diagnoses of exclusion such as idiopathic plexus neuritis could be made at the time. Fourth, the calculated cut-off ratio of 1.92 is dependent on the imaging parameters used on our scanner. Only a fully quantitative calculation by T2-relaxometry could overcome this limitation. We suggest that radiologists in other centers verify their cut-off values based on their own imaging parameters.

In conclusion, MRN was found to be a useful additional method in the diagnostic management of ulnar neuropathy of unclear localization and etiology. The results of this study encourage the systematic evaluation of MRN in other diagnostically challenging neuropathies.
